# Expression of Nek1 during kidney development and cyst formation in multiple nephron segments in the Nek1-deficient kat2J mouse model of polycystic kidney disease

**DOI:** 10.1186/s12929-014-0063-5

**Published:** 2014-07-17

**Authors:** Yumay Chen, Huai-Chin Chiang, Patricia Litchfield, Michelle Pena, Charity Juang, Daniel J Riley

**Affiliations:** 1 Department of Medicine, Division of Endocrinology, University of California, Gross Hall 1130, Irvine 92697, CA, USA; 2 Department of Medicine, Division of Nephrology, The University of Texas Health Science Center at San Antonio, San Antonio, USA; 3 University Transplant Center, The University of Texas Health Science Center at San Antonio, Medicine/Nephrology, MC 7882, 7703 Floyd Curl Dr, San Antonio 78229-3900, TX, USA; 4 Renal Research Division, South Texas Veterans Health Care System, Audie L. Murphy Division, 7703 Floyd Curl Dr, San Antonio 78229-3900, TX, USA

**Keywords:** Kidney development, Primary cilium, Centromere

## Abstract

**Background:**

Neks, mammalian orthologs of the fungal protein kinase never-in-mitosis A, have been implicated in the pathogenesis of polycystic kidney disease. Among them, Nek1 is the primary protein inactivated in kat2J mouse models of PKD.

**Result:**

We report the expression pattern of Nek1 and characterize the renal cysts that develop in kat2J mice. Nek1 is detectable in all murine tissues but its expression in wild type and kat2J heterozygous kidneys decrease as the kidneys mature, especially in tubular epithelial cells. In the embryonic kidney, Nek1 expression is most prominent in cells that will become podocytes and proximal tubules. Kidney development in kat2J homozygous mice is aberrant early, before the appearance of gross cysts: developing cortical zones are thin, populated by immature glomeruli, and characterized by excessive apoptosis of several cell types. Cysts in kat2J homozygous mice form postnatally in Bowman’s space as well as different tubular subtypes. Late in life, kat2J heterozygous mice form renal cysts and the cells lining these cysts lack staining for Nek1. The primary cilia of cells lining cysts in kat2J homozygous mice are morphologically diverse: in some cells they are unusually long and in others there are multiple cilia of varying lengths.

**Conclusion:**

Our studies indicate that Nek1 deficiency leads to disordered kidney maturation, and cysts throughout the nephron.

## Background

Animal models of polycystic kidney disease (PKD) have been invaluable in discovering and dissecting cellular and molecular pathways by which polycystic kidneys develop and by which progression of all types of PKD may be modified [1],[2]. Two murine models of polycystic kidney disease, the so-called kidneys-anemia-testes (kat and kat2J) mice [3], have been linked genetically to the locus encoding Nek1 protein kinase [4]. The kat and kat2J Nek1 mutations cause pleiotropic effects, including perinatal mortality in some pups, growth retardation, facial dysmorphism, abnormalities in the choroid plexus, PKD, and early death with or from progressive renal failure.

Nek1 is a mammalian ortholog of the fungal kinase NIMA (never in mitosis A in *Aspergillus nidulans*), which functions in DNA damage responses, regulates G2-M phase progression, and helps keep chromosome transmission to daughter cells faithful [5]-[10]. When cells are deficient in NIMA or in certain NIMA-related kinases, they undergo apoptosis because of major errors in chromosome segregation. Nek1 was identified as the first mammalian NIMA-related kinase. Partial cloning of the murine gene was accomplished using anti-phospho-tyrosine antibodies and reported more than fifteen years ago [11]. The kinase domain of Nek1, when expressed from bacteria, has dual serine-threonine and tyrosine kinase activity in vitro. Mechanisms by which Nek1 might normally function in a pathway to prevent the development of PKD are only beginning to be elucidated. We discovered that Nek1 is crucial for proper DNA damage responses and repair after injury [12]-[14], and that it limits injury in part by phosphorylating and controlling the VDAC1 channel that initiates mitochondrial apoptosis [15],[16].

Early reports described the cloning of murine Nek1 examined expression of mRNA by in situ analysis in murine gonads, and suggested that the abundant expression in both male and female germ cells was consistent with a role for Nek1 in meiosis [11],[17]. In situ analysis of kidney sections was not specifically reported. Tissue or cell-specific expression of the Nek1 protein was never reported, since no anti-Nek1 antibodies were available then, and the association of Nek1 with pleiotropic abnormalities including PKD was not made in mice until later [4].

As an important step in the characterization of how Nek1 may be involved in renal development and how its absence may lead to renal cystogenesis or cyst progression, we examined the expression pattern of Nek1 in normal, developing, and diseased mouse kidneys. Here we report that Nek1 is expressed most strongly in the distinct subset of renal epithelial cells. Its expression is developmentally regulated, such that it wanes as the kidney matures. Kidney development in kat2J/Nek1−/− mice is aberrant early, before the appearance of gross cysts: developing cortical zones are thin, populated by immature glomeruli, and characterized by excessive apoptosis of several cell types. Cysts in kat2J/Nek1−/− mice form postnatally in Bowman’s space as well as different tubular subtypes. Late in life, even kat2J/Nek1 +/− mice form renal cysts and the cells lining these cysts lack staining for Nek1. The primary cilia of cells lining cysts in kat2J/Nek1−/− mice are morphologically diverse: in some cells they are unusually long and in others there are multiple cilia of varying lengths. Our studies indicate that Nek1 deficiency leads to disordered kidney maturation, and to cysts formation throughout the nephron.

## Methods

### Kat2J mice and genotyping

C57Bl/6 J-*Nek1kat2J*+/−founder mice were obtained from The Jackson Laboratory (Bar Harbor, ME) and designated as the F0 generation. At F4, kat2J +/− males were mated with wild -type C57Bl/6 J females to generate F5. The resulting kat2J +/− mice were intercrossed until F8, when kat2J +/− males were mated with wild -type C57Bl/6 J females again. The kat2J colony was then maintained in this mating cycle. Genotyping for the *kat2J/Nek1* mutation was done as previously described [13],[14],[18], with one modification: the primers used in the PCR reaction were not labeled with an isotope. The PCR products were loaded onto a 9% acrylamide, 10% glycerol, 1x Tris/Borate/EDTA (TBE) buffer gel and subjected to electrophoresis for 2.5 hours at 40 W in 0.5x TBE buffer. The resulting gels were then stained with 4x Gel Red and analyzed with a UVP gel image system. All animal experiments were carried out in an ethical manner, in accordance with the protocols approved by the IACUC committees at University of Texas Health Science Center at San Antonio (protocol number: 01069B-34-03-A) and University of California, Irvine (protocol number: 2009-2899).

### Antibodies and lectins

Details of anti-Nek1 antibody generation and purification have been reported elsewhere [15],[18]. The anti-Nek1 antibodies used were rabbit polyclonals. We have characterized the specificity of these antibodies with western blots and immunohistochemistry, using appropriate controls (pre-immune serum, secondary antibodies alone, and lack of specific staining in kat2J/Nek1 −/− mouse kidneys) [15],[18]. Primary antibodies used in this study included rabbit anti-Nek1 (final concentration 10 μg/ml), anti-p84 mAb 5E10 (3 μg/ml) [19] (Genetex), anti-WT-1 (1:10 dilution, Santa Cruz), anti-PCNA (3 μg/ml, Santa Cruz, 1:250, Bethyl) and TUNEL reagents (Roche), anti-Tamm-Horsfall glycoprotein (1:50, Sigma-Aldrich), and anti-aquaporin-2 (1:100, Sigma-Aldrich). Peroxidase-labeled, secondary, anti-mouse, anti-rabbit and anti-Goat IgG antibodies and immuno-peroxidase-based ABC development kits were purchased from Vector Laboratories. Biotin-labeled *Lotus tetragonolobus* and *Dolichos biflorus* lectins (Sigma-Aldrich) were used at dilutions of 10 μg/ml and color was developed directly with the ABC peroxidase kit (Vector), i.e., without any antibodies.

### Tissue preparation and histology

Kidneys were harvested immediately after mice were sacrificed humanely. Specimens were fixed overnight in 10% neutral buffered formalin at 4°C. After progressive dehydration and embedding in paraffin, 3-μm sections were stained with Mayer’s hematoxylin and eosin reagents (Sigma-Aldrich). For immunohistochemical staining, 4-μm kidney or embryo sections on slides were deparaffinized with Histoclear (National Diagnostics) and rehydrated with graded ethanol. Immunoperoxidase-stained sections were counter-stained with methyl green to identify nuclei. For obtaining mouse embryonic tissues, timed matings of C57BL/6 mice were set up and embryos were harvested from sacrificed pregnant females at precise intervals thereafter. Post-coital day 0.5 was when the vaginal plug was identified in the impregnated female. Newborn mice were sacrificed within 24 hours of birth, and controls were always compared from the same litters.

### Scanning Electron Microscopy

Kidney samples were fixed in 4% neutral buffered formaldehyde/ 1% glutaraldehyde (pH 7.4) overnight at 4°C, then washed in 0.1 M phosphate buffer, post fixed in 1% Zetterqvist’s osmium tetroxide for 30 minutes, dehydrated with graded ethyl alcohols, and dried in a critical point dryer. Fractured sections were mounted, sputter-coated with gold and viewed with a Zeiss Leo 435 VP scanning electron microscope in the secondary electrons mode for topographical imaging [20]. The representative photomicrographs were taken with a digital camera.

## Results

### Nek1 expression in the normal mouse kidney

**Figure 1:**
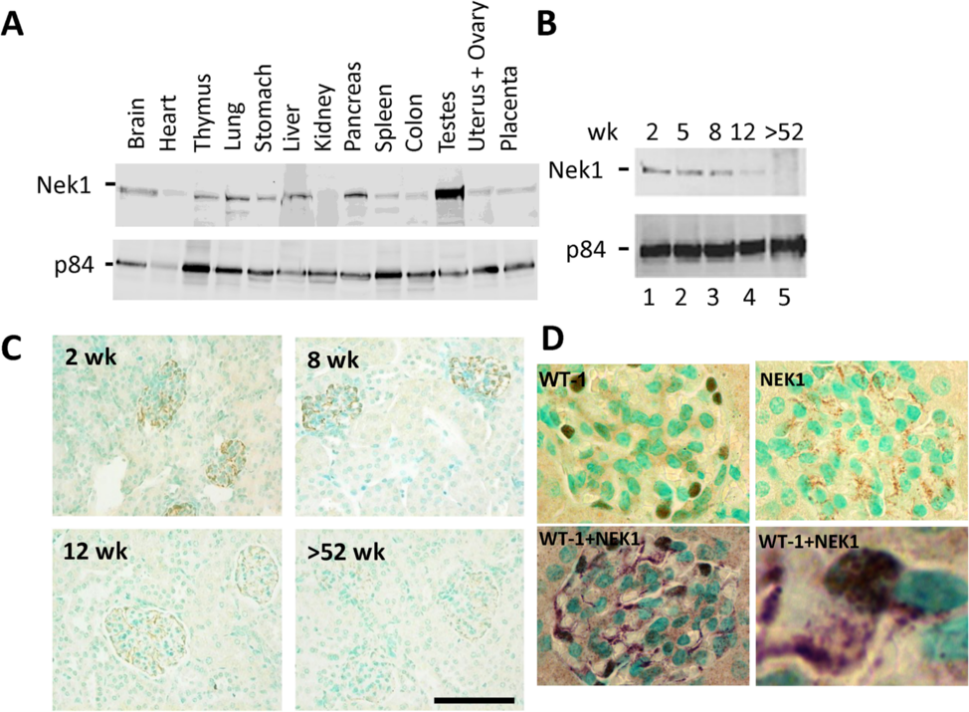
**Expression of Nek1 during kidney development. (A, B)** Nek1 is expressed ubiquitously but its expression diminishes as the mouse kidney matures. Western blots of tissue lysates. p84, a nuclear matrix protein, serves as a loading control. **(C)** Immunohistochemical staining for Nek1 in mouse kidneys at different ages, counterstained with methyl green to identify nuclei. Bar, 100 μm. **(D)** Nek1 is expressed in the cytoplasm of glomerular epithelial cells. Formalin-fixed, paraffin-embedded section was dual stained with rabbit anti-Nek1 (developed with brown- (upper corner), or purple- (lower panel) conjugated anti-rabbit IgG secondary antibody) and mouse monoclonal anti-WT-1 (developed with brown-conjugated anti-mouse IgG secondary antibody). Cytoplasmic staining for Nek1 is evident in the same cells with nuclear staining for WT-1.

We generated anti-Nek1 antibodies and characterized them extensively for their specificity [15],[18]. To determine which organs express Nek1, we looked at Nek1 protein from adult mouse tissue lysates by immunoblotting analysis. Nek1 is expressed in all organs examined, but its expression is not very abundant in the mature kidney compared to other organs (Figure 1A). Other proteins functionally inactivated in PKD, including polycystins, are likewise expressed only weakly in the normal adult kidney, and only in a subset of kidney cells [21]-[26]. To know whether Nek1 expression is regulated during kidney development and maturation, we also examined protein lysates from kidneys of mice at different ages. The expression of Nek1 decreases significantly as the post-natal kidney matures (Figure 1B).

To clarify observations made by immunoblotting proteins separated from whole kidney lysates, and to determine which subsets of kidney cells express Nek1 most abundantly, we further examined mouse kidney sections by immunohistochemistry (IHC) (Figure 1C). Anti-Nek1 antibodies strongly stained glomerular epithelial cells (podocytes). They also stained several subtypes of tubular epithelial cells less strongly, including those that comprise proximal tubules. Nek1 expression in glomerular epithelial cells, although it also waned as the kidneys aged, was relatively strong in both immature and mature kidneys. Expression of Nek1 in tubular epithelial cells, in contrast, was much more prominent in the immature mouse kidney than in the adult kidney. The specificity of the immunostaining was assured by repeating the experiment with pre-immune rabbit serum (not shown and [18]). These IHC results are consistent with the results observed from immunoblotting of whole kidney lysates from mice at different ages: Nek1 expression diminishes as the kidney and its epithelial subtypes mature. Co-staining of Nek1 in the cytoplasm and WT-1 in the nuclei of the same cells confirmed that Nek1 expression in glomeruli is the strongest in podocytes (Figure 1D).

### Nek1 expression in the embryonic mouse kidney

**Figure 2:**
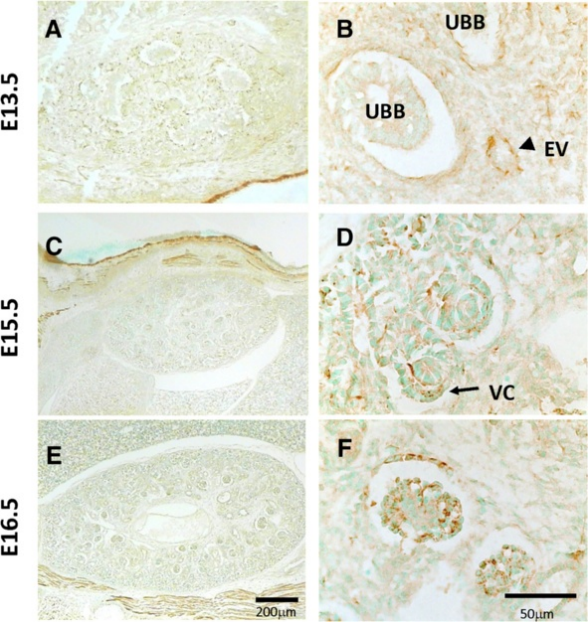
**Nek1 expression in wild type mouse embryonic kidneys.** Sections from embryonic mouse kidney at E13.5, E15.5 and E16.5 were subjected to immunohistochemistry analysis for Nek1 expression. **(A, C, E)** Low power photomicrographs (original magnification l0x, scale bar, 200 μm). **(B, D, F)** Higher power detail of the same kidney sections (original magnification 400x, bar, 50 μm). UBB, ureteric bud branch; EV, epithelial vesicle induced by ureteric bud; VC (thick arrow), vascular cleft of developing S-shaped proximal nephron.

We also examined the expression of Nek1 by IHC analysis in developing mouse kidneys. At post-coital embryonic day 13.5, when a recognizable kidney can first be identified, nephrogenesis proceeds by induction of metanephric mesenchyme from branches of the ureteric bud. Individual nephrons, from the glomerular capillary to the distal tubule, coalesce from induced mesenchyme, which sequentially forms in a centripetal pattern into comma- and S-shaped bodies in the more mature, inner regions [27],[28]. Nek1 expression is relatively strongest in the developmentally primitive nephrogenic zones of the subcapsular cortex (Figure 2). Mesenchymal cells induced by branches of the ureteric bud to differentiate into epithelial vesicles express Nek1 most abundantly (Figure 2, panel B), as do cells at distinct regions of the lesser curvatures and bases of comma- and S-shaped bodies (Figure 2, panel D). These latter regions at and near the vascular cleft are precursors of glomerular and proximal tubular epithelial cells [27],[28]. In the more mature yet still primitive glomeruli in the future juxtamedullary region of the kidney, Nek1 expression is evident in the cytoplasm of some tubular epithelial cells, but is strongest in parietal and visceral glomerular epithelial cells (Figure 2, panel F). This pattern in nephrons more advanced in their development is what would be expected in later stages from the Nek1 expression pattern in early postnatal kidneys. Taken together, the IHC data in normal developing mouse kidneys show that Nek1 is expressed most prominently in future glomerular and tubular epithelial subtypes. The data suggest that Nek1 may be important during stages of kidney development when epithelial cells are still cycling, rapidly synthesizing and repairing DNA, differentiating, or undergoing programmed cell death.

### Excessive apoptosis in the kat2J mouse kidney

**Figure 3:**
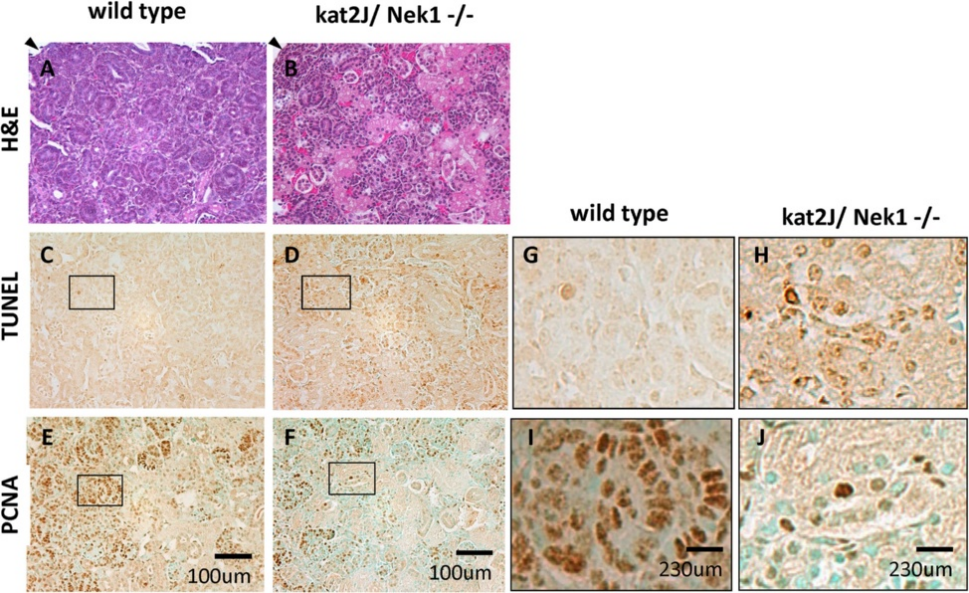
**Thin cortical zone, increased cell death, and decreased proliferation in newborn kat2J/Nek1 −/− kidneys. (A, B)** Hematoxylin and eosin (H&E)-stained wild type **(A)** and kat2J/Nek1−/− **(B)** kidney sections. Arrowheads in the upper right corner of photomicrographs of hematoxylin and eosin **(H&E)**-stained sections indicate the renal capsule. Note thinner, more disorganized, subcapsular, cortical zone in the representative kat2J/Nek1 −/− kidney. **(C, D, G, H)** Excessive apoptosis in Kat2J/Nek1−/− kidneys. Kidney sections from wild type **(C&G)** and kat2J/Nek1−/− mice **(D&H)** were analyzed by TUNEL assay. Note more TUNEL-positive, apoptotic cells in the kat2J/Nek1−/− kidney). **(E, F, I, J)** The proliferation status of cells in wild type **(E&I)** and kat2J/Nek1−/− **(F&J)** kidneys was analyzed by PCNA expression. Note relatively fewer PNCA-positive, proliferating cells in the kat2J/Nek1 −/− kidney. Bar, 100 μm in panels **A-F**; 230 μm in panels **G-J**.

If Nek1 is important for kidney development, as suggested by its expression pattern, then kidney maturation should be delayed or otherwise abnormal in Nek1-deficient kat2J mice. To examine this possibility, we compared the kidneys of Nek1-deficient kat2J mice to those of age- and sex-matched, wild type littermates. Previous publications characterizing the phenotypes of kat and kat2J mice reported that polycystic kidneys were observed late in mouse development, at least 1 month after birth in the more severely affected kat2J strain [3]. We found a significant difference between the kidneys of kat2J/Nek1 −/− mice and wild type mice, however, as early as hours after birth. Subcapsular nephrogenic zones in kat2J/Nek1 −/− kidneys are thinner and less differentiated (Figure 3A & B). Many kat2J/Nek1 −/− tubular epithelial cells in H&E sections appear eosinophilic and vacuolated, as dying cells would. Staining with TUNEL reagents confirmed that cells in tubules and in primitive glomeruli die by apoptosis or necrosis at much higher rates in kat2J/Nek1 −/− kidneys (Figure 3C, D, G & H). Staining for proliferating cell nuclear antigen (PCNA) also showed fewer cells in the S-phase of the cell cycle in the developing kat2J/Nek1 −/− kidneys (Figure 3E, F, I & J).

### Delayed maturation in the kat2J mouse kidney

**Figure 4:**
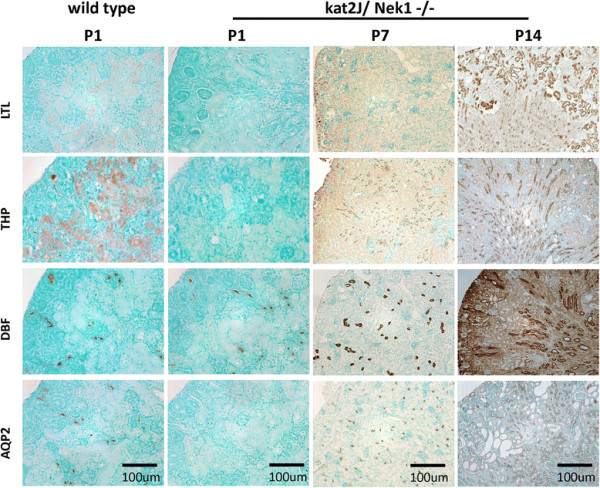
**Delay in kidney development and maturation in kat2J/Nek1−/− mice.** Aberrant and delayed maturation of metanephric mesenchyme-derived tubules in the cortex of kat2J/Nek1 −/− kidneys. Sections of kidneys from newborn mice were immunostained with lectins marking proximal tubules (LTL) and collecting tubules (DBF), or with primary antibodies recognizing loops of Henle (THP) or collecting ducts (AQP2). They were then developed directly with immumoperoxidase or indirectly after incubation with secondary antibodies (THP and AQP2), and counterstained with methyl green. Bar, 100 μm. Sections from postnatal day 7 and 14 (P7 and P14) kat2J/Nek1−/− mice were immunostained with the same lectins or primary antibodies. They were then developed directly with immumoperoxidase or indirectly after incubation with secondary antibodies, as above, and counterstained with methyl green. Bar, 100 μm.

Tubular maturation is also abnormal in newborn kat2J/Nek1 −/− mice. Of the 7 newborns kat2J/Nek1 −/− mouse kidneys examined (from 3 litters), only one showed positive staining with a marker for mature proximal tubules (*Lotus tetragonolobus* lectin, LTL). The following tubular subtypes did not stain for any markers: Tamm-Horsfall glycoprotein (THP) for loops of Henle or aquaporin-2 for mature collecting ducts (Figure 4). Staining for a marker of collecting tubules, *Dolichos biflorus* lectin (DBF), in contrast, was positive in kat2J/Nek1 −/− kidneys in a similar pattern when compared to wild type kidneys. In the kidneys of kat2J/Nek1−/− mice at postnatal day 7 (n = 6, from 3 litters) and postnatal day 14, mature proximal tubules, distal tubules, and collecting ducts were identified by staining with markers (Figure 4).

**Figure 5:**
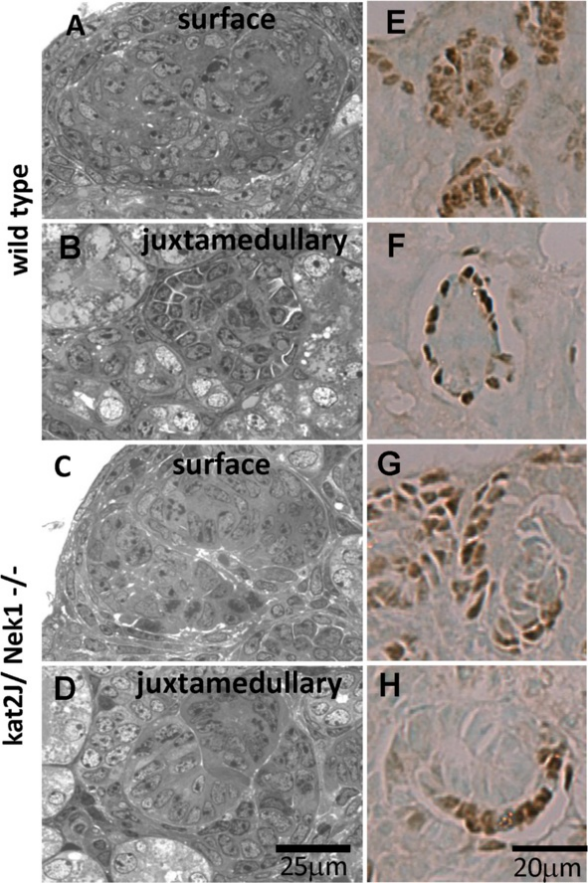
**Delayed glomerular maturation in newborn kat2J/Nek1 −/− kidneys.** Epoxy-embedded, toluidine blue stained sections of subcapsular (surface) and deeper (juxtamedullary) glomeruli from representative, littermate, wild type **(A, B)** and kat2J/Nek1 −/− **(C, D)** kidneys. Compare the more mature, wild type, juxtamedullary glomerulus in B, to the kat2J/Nek1−/− juxtamedullary glomerulus still in S-shaped form in D. Bar, 25 μm. Immunocytochemical staining of formalin-fixed, paraffin-embedded sections from the same kidneys for WT-1, a nuclear marker for metanephric mesenchyme and podocytes **(E-H)** confirmed that in kat2J/Nek1−/− kidneys, nascent juxtamedullary glomeruli were still in S-shaped forms **(G, H)**. At the same time, nascent juxtamedullary glomeruli in kidneys of sex-matched, wild type, littermate mice have progressed to more mature architecture, with WT-1 staining in a ringed podocyte pattern **(E, F)**. Bar, 20 μm.

In addition to the delay in tubular development, glomerular development in kat2J/Nek1 mice appears to be delayed as well (Figure 5): more than 50% of deeper, juxtaglomerular glomeruli are still in comma-shaped stages when similar glomeruli in wild type littermates have already progressed to a more mature stage. Such differences were evident by examining glomerular architecture in ultrathin, epoxy-embedded kidney sections stained with toluidine blue, and IHC staining for WT-1, a nuclear marker of metanephric mesenchymal cells and podocytes. All of the histological and immunohistochemical results suggest aberrant maturation of glomeruli and of several different tubular subtypes, especially those derived from metanephric mesenchyme, in maturing kat2J/Nek1 −/− kidneys. These delays in maturation occur well before the kidneys develop recognizable cysts.

### Cysts develop from multiple different epithelial types in the nephron, from Bowman’s space to collecting ducts

**Figure 6:**
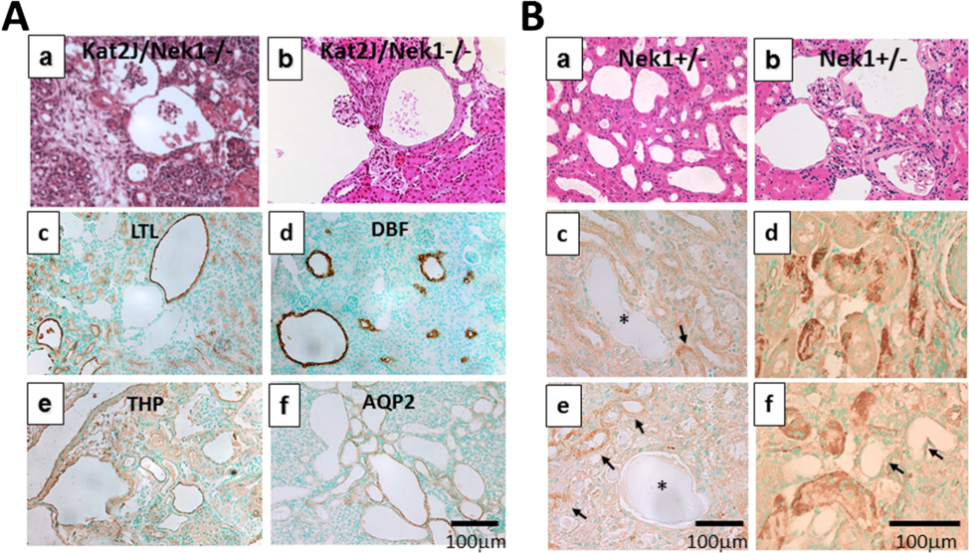
**Cyst formation in kat2J mice. (A)** Cysts in kat2J/Nek1 −/− kidneys form in different parts of the nephron and from multiple tubular subtypes. Representative H&E-stained sections of a kidney from a 1-day-old mouse (a, original magnification 40x) and 21-day-old mouse (b, original magnification 40x; bar, 100 μm) show tubular and glomerular cysts. (c-f) Immunohistochemical staining for proximal tubules with *Lotus tetragonolobus* lectin (c, LTL), loops of Henle with Tamm-Horsfall glycoprotein (e, THP), collecting tubules with *Dolichos biflorus* lectin (d, DBF), and other collecting ducts with aquaporin-2 (f, AQP2). Bar, 100 μm. **(B)** Cystic disease in kat2J/Nek1+/− mice. (a, b) Cysts develop focally in kat2J/Nek1+/− old adult mice (age 415 days). (c, d) Different sections from the same aged different mouse show patchy upregulation of Nek1 in some tubules (arrows) but loss of Nek1 staining in cystically dilated tubules (asterisks). (e, f) In representative sections from a kat2J/Nek1 +/− littermate, Nek1 expression is upregulated in some tubules but minimal or absent in other, cystically dilated tubules. Bar, 100 μm for all panels.

Unlike observations made in a previous report [3], we found cysts in the kidneys hours after birth in the inbred kat2J/Nek1 −/− mice (Figure 6A panel a). Glomerular cysts expanding the Bowman’s space and tubular cysts are more evident at 2 weeks of age (Figure 6A, panel b). Unlike the previous report, in which most kat2J/Nek1−/− mice survived past 7–8 months of age, most of the kat2J/Nek1−/− mice in our inbred colony did not survive past 20 days old. Staining with lectins or proteins that have expressions limited to certain tubular subtypes indicate that the polycystic kidney disease in kat2J/Nek1 −/− mice develops from multiple tubular subtypes, including proximal tubules, loops of Henle, and collecting tubules or collecting ducts (Figure 6A, panels c-f), as well as from glomerular epithelial cells (Figure 6A, panel b). In this respect, the polycystic kidney disease that develops in kat2J/Nek1 −/− mice is much like human autosomal dominant PKD, in which cysts can form from all parts of the nephron [29],[30].

### Focal cysts develop in older kat2J/Nek1 +/− mice

The PKD phenotype in the kat and kat2J mice is thought to have a recessive pattern of inheritance according to the previous report [3]. In other words, only mice with homozygous Nek1 mutations develop the disease. We examined heterozygous kat2J/Nek1 mice long into their life spans, however, and discovered that even these mice develop focal cystic disease as they age. Whereas inbred kat2J/Nek1 −/− mice develop morphologically detectable PKD early in their lives, almost universally before the age of 3 weeks, most of their Nek1+/− littermates (55 of 81, or 68%) developed a more limited form of cystic disease after the age of 1 year (Figure 6B, panels a & b). Damage to the kidney with ischemia-reperfusion injury can accelerate this focal or limited PKD ([31] and manuscript in preparation). The cystic disease was never seen in similarly treated wild type littermates, even after ischemia-reperfusion injury [31]. Although expression of Nek1 in tubular epithelial cells is decreased overall in older mice compared with younger mice, many tubular epithelial cells in aged kat2J/Nek1 +/− kidneys have up-regulated Nek1 expression in a patchy distribution (Figure 6B, panels c & d). Other tubular epithelial cells in kat2J/Nek1 +/− mice, especially those that line nascent cysts or cystic, dilated tubules, don’t stain for Nek1 at all (Figure 6B, panels e & f). These results suggest that the loss of Nek1 expression may be important, either etiologically in cyst generation or as a consequence of early cyst development. Stochastic inactivation of Nek1 may be required for cystogenesis in the kat2J model.

### Primary cilia are morphologically diverse in cyst-lining epithelial cells

**Figure 7:**
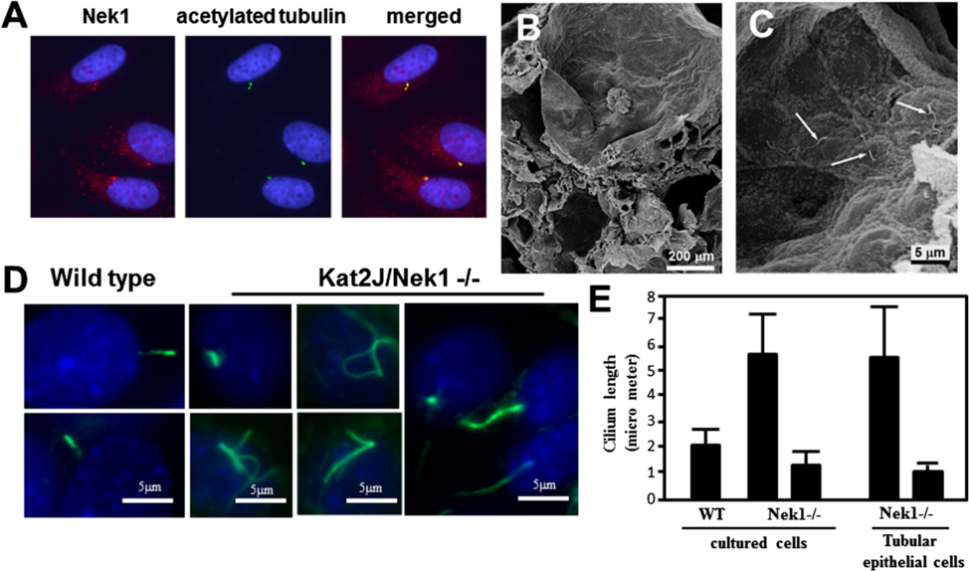
**Primary cilia in kat 2 J/Nek1 −/− tubular epithelial cells. (A)** In cultured HK2 human renal tubular epithelial cells, a portion of cellular Nek1 localizes at the base of primary cilia (or the centrosomes). Blue fluorescence from DAPI identifies nuclei, red identifies immunostained Nek1, and green identifies the primary cilium immunostained for acetylated tubulin. **(B)** Representative scanning electron micrograph of kidney from a kat2J/Nek1 −/− mouse with advanced PKD, showing a glomerular cyst and multiple tubular cysts. **(C)** Morphologically normal but long (5–9 μm) primary cilia are seen in tubular epithelial cells lining a cyst in the same kat2J/Nek1 −/− mouse kidney. **(D)** Wild type renal tubular epithelial cells cultured and passed multiple times have mostly normal and single primary cilia, stained at their base with a fluorescent green-tagged antibody recognizing acetylated tubules. Kat2J/Nek1 −/− cells from sex-matched littermates, cultured in identical conditions, had more variable primary cilia, including many cells with abnormally short, long, and multiple cilia. DAPI stains nuclei blue. Bar, 5 μm. **(E)** Cilia length in kat2J/Nek1 −/− kidney and cells. The length of cilia was measured using an image analysis program (AxioVision Rel 4.6, Carlo Zeiss). The mean lengths of the cilia are plotted with standard deviations. The number of cilia analyzed in images of cultured cells were n = 30 for wild-type cells, n = 25 for long cilia, and n = 16 for short cilia at2J/Nek1−/− cells. For the scanning EM images of kat2J/Nek1 −/− kidneys, n = 6 for long cilia, n = 10 for short cilia.

Disturbances in the morphology or function of primary cilia are currently thought to be fundamental to the pathogenesis of PKD, in many animal models as well as in human autosomal recessive and autosomal dominant PKD [2],[32]. Using the anti-Nek1 antibodies we developed, we and other researchers have shown that a portion of Nek1, like many other PKD-associated proteins, localizes to the primary cilia in normal renal tubular epithelial cells [33]. We also found Nek1 to co-localize with acetylated tubulin, in particular at the base of the primary cilia or at centrosomes in interphase HK2 human renal tubular cells or renal tubular epithelial cells (RTEs) cultured primarily (without any transformation) from wild type mice (Figure 7A). In primarily attached RTEs cultured from wild type littermates of kat2J/Nek1 −/− mice, the staining pattern for acetylated tubulin was similar to the pattern observed in wild type mice. In kat2J/Nek1 −/− RTEs that had been passed for several generations in culture, however, primary cilia were morphologically diverse compared to those of similarly passed wild type RTEs. Many kat2J/Nek1 −/− cells had unusually long cilia, and some had multiple cilia (Figure 7D). Scanning electron micrographs of kidneys from kat2J/Nek1 −/− reiterated previous data that cysts formed in Bowman’s space and in multiple tubular segments (Figure 7B). They also allowed visualization of cilia in the tubular epithelial cells lining cysts, which appeared morphologically normal, but are usually longer than expected (Figure 7C). The length of the cilium is subject to biological regulation. Different cell types possess cilia of different average lengths. Studies from mouse suggest the mean ciliary length in normal kidney tubule is 2.2-2.3 μm [34]. In our cultured wild-type renal tubular epithelial cells, the mean ciliary length is 2.1 μm, which is similar to the length in the other report. In the kat2J/Nek1−/− kidney, the mean, abnormally long ciliary length is 5.6 μm and the short ciliary length 1.10 μm. In the cultured kat2J/Nek1−/− cells, the mean long ciliary length is 5.6 μm and the short ciliary length 1.4 μm, very similar to the lengths found in the mutant kat2J/Nek1−/− kidneys by scanning electron microscopy (Figure 7E). Thus some cilia are longer than normal and others are shorter, and some cells have more than one primary cilium. The length of the cilia is important for the ability of cells to respond properly to stress, directional flow, or other environmental stimuli. The abnormal cilial length in Kat2J/Nek1−/− cells and kidneys may result in kat2J/Nek1−/− cells that cannot sense or respond to stress correctly, and that cells dividing along tubules during development or after injury may not populate in a proper planar direction. Such misdirected cell polarity and migration could contribute to the generation of cysts.

## Discussion

In this study, we explored the expression pattern of Nek1, which is mutated and inactived in the kat and kat2J mouse models of PKD. Like polycystins 1 and 2 (PC1 and PC2), Nek1 appears to be developmentally regulated. Expression is highest during the middle stages of the embryonic kidney development, and specifically in cells destined to become glomerular and proximal tubular epithelial cells. Expression of Nek1 in proximal tubular cells diminishes within weeks after birth in the mouse, whose kidney still grows and matures postnatally. Expression of Nek1 in glomerular epithelial cells, although it also diminishes with kidney maturation, remains relatively strong compared with other kidney cells well into adult stages. Why Nek1 expression seems to be strongest in podocytes, and to a lesser extent in tubular epithelial cells, is not entirely clear. Perhaps these cells have specific replicative steps that require Nek1 for efficient DNA repair [12],[13], or perhaps they need Nek1 at primary cilia/centrosome complexes for uniquely sensing directional proliferation as glomeruli coalesce and as tubules expand and elongate. Such functions would be required less in mature glomeruli, the components of which are no longer rearranging as actively by cell proliferation and apoptosis as they were in earlier stages, and in tubules after they no longer need to elongate. In such a scenario, Nek1 expression would increase again in cells after injury in post-natal stages, because the cells would again require new DNA synthesis, checking and repair of damaged DNA, and mitosis with directional proliferation to repopulate glomerular epithelial spaces and renal tubular lumens. In cells lining non-cystic tubules in Nek1 +/− kidneys, we found that Nek1 expression was up-regulated after ischemic injury (data not shown, [31]).

Although there is controversy about some of the antibody specificities used to describe the distribution pattern of polycystins in mouse embryonic and adult kidneys, immunohistochemical studies on PC1 and PC2 expression and in situ hybridization techniques used to observe the expression of *PKD1* and *PKD2* mRNA transcripts have found consistent patterns in many cases. The similarities and differences in the expression patterns of polycystins and Nek1 are interesting to note. First, Nek1 is expressed prominently in podocytes; PC1 and 2 are not. *PKD1* expression during embryonic kidney development first appears by mRNA in situ analysis close to birth in the distal tubules and relatively late in differentiating proximal tubules. *PKD2* mRNA, in comparison, is more diffusely expressed during all stages of nephron development. [21],[24] Expression of both *PKD1* and *PKD2* mRNA by in situ hybridization and PC1/2 proteins by immunohistochemical staining diminish significantly in the mature kidney compared to the still developing kidney. These findings prompted speculation that polycystins have roles primarily related to differentiation and organization of tubular architecture during nephrogenesis [21]-[24],[26]. Inactivation of either polycystin-1 or −2, has also been shown to lead to aberrant renal tubular cell proliferation and apoptosis [21]-[24],[26],[35],[36]. Nek1 may have similar functions, or unique functions in the same developmental pathways as the polycystins during development and after injury.

The recent demonstration that Nek1 interacts with and phosphorylates TAZ, an adaptor protein in the E3 ubiquitin ligase complex, to modulate PC2 protein level [37] provides another potential mechanism by which Nek1 inactivation may lead to polycystic kidney disease. In Nek1−/− cells, TAZ becomes unphosphorylated and fails to target PC2 for degradation, rsulting in abnormal accumulation of PC2 and affecting its role in ciliogenesis. Transgenic mouse models that overexpress PC2 argue against upregulation of PC2 protein expression as the main reason to explain PKD in kat2J Nek1−/− mice. In both PC2 transgenic mouse models, the mice develop small cysts as early as 8 month after birth, and more cysts by 18 months of age [38],[39]. In the kat2J Nek1−/− mice, cysts develop as early as 5–14 days. Additional mechanisms by which Nek1 plays in cytogenesis need to be explored.

Our data clearly indicate that Nek1 deficiency causes abnormalities early in kidney development, before the development of grossly evident glomerular or tubular cysts. The early abnormalities include excessive apoptosis and diminished proliferation. These could lead to reduction in net nephron mass as well as to the niduses for cysts, where expanding Bowman’s capsules or elongating tubules develop weak regions at which they might herniate or focally expand the tubular lumen. These abnormalities therefore provide insight into the pathogenesis of cysts in the Nek1/kat2J model of PKD. They also illustrate a theme common for many types of polycystic kidney disease: the cystic phenotype requires or is greatly exacerbated by cellular proliferation and mitosis.

Several animal models of PKD, including conditional inactivation of murine Pkd1, kif3a (a kinesin motor component important for directional transport in primary cilia), or other proteins, have been shown to develop the polycystic phenotype only when the gene is inactivated during kidney development or postnatally after the kidney is injured and allowed to repair [40]-[42]. If such proteins are conditionally inactivated after full kidney maturation and if the adult kidneys are not injured such that renal cells are not forced to divide, then cysts rarely develop. The key requirement for cystogenesis seems to be the proliferation of epithelial cells, perhaps accompanied by inaccurate mitosis and/or cytokinesis, and altered planar polarity in daughter cells moving along a specific vector. Some researchers have suggested that manifestation of autosomal dominant PKD requires not only two genetic “hits” to *PKD1*, but a “third hit”, i.e., aberrant mitosis during embryonic or neonatal kidney development, or injury with repair [40],[43],[44].

We speculate that the same requirement for mitosis would be necessary if Nek1 were conditionally inactivated in experimental mice. Inactivation of Nek1 itself, however, since it leads to mitochondrial cell death in the presence of minimal stress, and defective DNA damage sensing and repair [15],[16], could propagate injury to nearby tubules, even without additional ischemia-reperfusion injury. The requirement of cellular proliferation for the manifestation of cystic disease suggests that defects in mitosis are fundamental to the pathogenesis of renal cysts. We have ascribed roles for Nek1 to ensure proper primary cilium-centrosome functions in dividing cells. Aberrant mitoses result when Nek1 is inactivated [12],[18].

Some of our findings differ from those previously reported. Early reports characterizing the kat and kat2J strains suggested that renal cysts do not form until postnatal developmental stages, and that they do not progress to end stages until 3 to 9 months after birth [3],[4]. We used the same kat2J strain developed at The Jackson Laboratory as the one reported by Uphadya et al. Perhaps we observed more accelerated pleiotropic pathology in our more inbred colony on the C57Bl/6 J genetic background. Even with subsequent out-breeding to wild type C57Bl/6 J mice, we still found earlier development of renal cysts than previously reported in Nek1 −/− kat 2 J mice. There may be an anticipation phenotype in after multiple Nek1/kat2J +/− x +/− matings, owing to the effects of heterozygosity for the *Nek1* gene. Nek1 haplo-insufficiency does result in impaired responses to DNA damage [14], which might also help to explain our observation that older Nek1 +/− mice develop focal cystic disease.

Our data in cultured renal tubular epithelial cells and fibroblasts has previously suggested that Nek1 upregulation may be a protective response to DNA and cellular damage [12],[13],[15]. After ischemic/oxidative kidney injury, which causes DNA damage, Nek1 expression is upregulated, and a portion of cellular Nek1 moves from cytoplasm to the foci of DNA damage in the nucleus. If the damage is sublethal, then Nek1 moves back to its normal cytoplasmic location and the cells survive [12],[15]. In the present study, we show in vivo evidence to support this same hypothesis, i.e., that Nek1 expression is upregulated, perhaps as a protective response to cellular and DNA damage. The expression of Nek1 increases after ischemia-reperfusion injury in tubular epithelial cells that aren’t cystic, but seems to be down-regulated or absent in renal tubular epithelial cells that line nascent renal cysts in Nek1/kat2J heterozygotes (Figure 6). These results suggest that stochastic inactivation of Nek1 is required for cyst formation in the kat2J mouse model of PKD. Aberrant planar polarity, mitotic catastrophes, and excessive apoptosis would result if dividing cells that line Bowman’s spaces or renal tubules could not properly repair injured DNA in the setting of Nek1 deficiency. Aberrant mitoses that were not lethal by the next cell division might result in inequitable distribution of chromosomes to daughter cells. Our previous observations in cultured Nek1/kat2J −/− cells have proven chromosomal instability in Nek1-deficient cells [18].

Additional observations in the current study have shown that primary cilia, which have the same bases as the centrosome complexes, are morphologically variable in Nek1 −/− cells. Most of the identifiable cilia in renal tubular cells observed by scanning electron microscopy in Nek1/kat2J −/− renal cysts (and in primary Nek1−/− tubular cells before propagation in culture) were relatively normal and uniform in appearance, although somewhat longer than expected. After such cells were made to divide several times in culture, however, primary cilia seemed to become more variable in length and even in number. These primary cilium changes would also fit with previous findings from our lab showing that Nek1 −/− cells suffer mitotic errors in surviving cells [12]. In this case, the mitotic errors manifest as bizarre and heterogeneous primary cilia. We suggest that improperly regulated mitosis and cytokinesis are the primary problems in Nek1 deficiency, and probably in other forms of PKD as well.

At least 11 mammalian NIMA-related proteins have been identified to date. They all have significant homology in their N-terminal kinase domains, but widely divergent regulatory domains with distinct binding characteristics and presumably distinct kinase substrates [5]. Nek8 is particularly noteworthy in comparison with Nek1, because the gene encoding it has also been mapped to a spontaneous mutation associated with PKD in the juvenile polycystic kidney (jck) mouse strain [45]. Immunohistochemistry has shown normal localization of Nek8 in the apical cytoplasm of inner medullary collecting tubule cells but mislocalization in the collecting tubules of Nek8/jck −/−mouse kidneys. Expression of a dominant negative form of Nek8 in cultured tubular epithelial cells results in disordered actin cytoskeleton and in multiple nuclei. These findings and the unique RCC1 repeats in the Nek8 protein, which are important for chromosome condensation in other proteins, have suggested that Nek8 might serve an essential function in regulating the cytoskeletal structure and regulate chromosome segregation in a specific subset of renal cells.

Nek1, by virtue of the unique protein interactions and expression pattern, may serve a distinct but related function in different kidney cell types. In contrast to the expression of Nek8, primarily in collecting tubules and derived embryologically from ureteric buds, Nek1 expression is greatest in developing proximal tubules and glomerular epithelial cells, which derive from metanephric mesenchyme. It is tempting to speculate that Nek1 and Nek8 may serve similar functions in distinct renal epithelial cell types derived from different embryonic origins. In Nek1/kat 2 J −/− kidneys, however, we did observe some cysts stained with markers that are usually restricted to the collecting tubules and ducts (aquaporin-2 and DBF lectin). Nek1 deficiency must therefore have some effects on the development of cysts derived from the tubules of ureteric bud origin. Alternative explanations for our observation that some cyst-lining renal tubular epithelial cells stain with DBF lectin and aquaporin-2 are that these markers are not entirely specific for collecting tubules, or that the incompletely differentiated cells lining cystic tubules don’t stain exactly like normal tubules, such that they cannot be classified as collecting tubules or ducts. While data suggests that lectins may not always identify tubular subtypes with absolute certainty [46]-[48], they have been used accurately in studies of polycystic kidneys in the same way we used them here, to determine tubular cell types from which cysts derive in PKD [49]-[51].

Taken together the data presented here, the pattern of Nek1 expression during development and after kidney injury and the finding that renal cysts in the Nek1 −/− and +/− models derive from many different epithelial cells types along the nephron, indicate that the kat2J mouse is relevant for studying the pathogenesis of several forms of PKD. We show here for the first time that Nek1 +/− mice develop a mild form of cystic kidney disease late in life. Since cyst-lining cells lose expression for Nek1, we suggest that stochastic inactivation of Nek1 is required for the manifestation of the PKD phenotype, as it is in humans and animals with heterozygous germ line mutations in *PKD1* or *PKD2* to manifest autosomal dominant PKD. Lastly, we observed that the morphology of primary cilia in Nek1 −/− renal tubular cells changes and becomes more variable as the cells serially divide. This adds credence to the hypothesis that polycystic kidney disease is a problem that stems fundamentally from abnormal functions of the primary cilium-centrosome complex, including abnormalities that arise from imperfect mitosis and inequitable distribution of centrosomes and chromosomes.

Aberrant apoptosis is an unique and cardinal feature of cystic and non-cystic kidney cells in progressive human ADPKD [52]. Based on the roles of Nek1 in DNA sensing, responding to, or repairing DNA and cellular damage, and regulating renal cell apoptosis after injury[12]-[16],[18],[53], Nek1 is thereby important for regulating renal cell apoptosis after injury. In this scenario, Nek1 expression is upregulated as a defective response [14]. Increase in Nek1 abundance and kinase activity may be needed specifically in injured cells before they recover; such response would allow sub-lethally injured cells to sense and/or repair damage to DNA and other cellular structures more efficiently [53]. When Nek1 is mutated or when its upregulation is otherwise insufficient, cells in the kidney would more likely die aberrantly or fail to proliferate when they normally should after injury. The abnormal length of cilia found in the Kat2J/Nek1−/− kidney and cells also suggests that Nek1 deficient cells may not be able to sense the environmental stress and response accordingly. Such a series of events may begin to explain how Nek1 deficiency leads to polycystic kidney disease in mice, through a mechanism that involves accelerated and aberrant apoptosis in the setting of endogenous or acquired DNA and cellular damage.

## Conclusions

In the report, we examined the expression pattern of Nek1 and characterized the renal cysts that develop in kat2J mice. We showed that Nek1 is expressed in all murine tissues examined. Its expression in kidney is developmentally regulated: in the embryonic kidney, Nek1 expression is most prominent in cells that will become podocytes and proximal tubules, in the postnatal kidney, Nek1 expression decreases as the kidneys mature, especially in tubular epithelial cells. Kidney development in kat2J/Nek1−/− mice is aberrant early, before the appearance of gross cysts: developing cortical zones are thin, populated by immature glomeruli and characterized by excessive apoptosis of several cell types. Cysts in kat2J/Nek1−/− mice form postnatally in the Bowman’s space as well as different tubular subtypes. Late in life, kat2J/Nek1 +/− mice form renal cysts and the cells lining these cysts lack staining for Nek1. The primary cilia of cells lining cysts in kat2J/Nek1−/− mice are morphologically diverse: in some cells they’re unusually long while in others, there are multiple cilia of varying lengths. Our studies indicate that Nek1 deficiency leads to disordered kidney maturation and cysts throughout the nephron.

## Abbreviations

PKD: Polycystic kidney disease

NIMA: Never-in-mitosis A protein kinase

Nek1: Never-in-mitosis A related protein kinase

Kat: Kidneys-anemia-testes

PCNA: Proliferating cell nuclear antigen

LTL: *Lotus tetragonolobus* lectin

THP: Tamm-Horsfall glycoprotein

DBF: *Dolichos biflorus* lectin

PKD1: Polycystic kidney disease gene 1

PKD2: Polycystic kidney disease gene 2

PC1: Polycystin 1, protein product of PKD1

PC2: Polycystin 2, protein product of PKD2

TBE: Tris/Borate/EDTA buffer

VDAC: Voltage dependent anionic channel

PCR: Polymerase chain reaction

TUNEL: Terminal deoxynucleotidyl transferase (dUTP) nick end labeling

IHC: Immunohistochemistry

## Competing interests

The authors declare that they have no competing interests.

## Authors’ contributions

YC conceived, designed, and coordinated the study, acquired, analyzed, and interpreted data, and helped draft the manuscript. HCC, MP, and PL performed or helped interpret the histological sections and animal handling. CJ helped with the cilia analysis and drafted the final manuscript. DJR conceived, designed, acquired, analyzed, and interpreted data, and drafted the manuscript. All authors read and approved the final manuscript.
